# Oganesson Is a Semiconductor: On the Relativistic Band‐Gap Narrowing in the Heaviest Noble‐Gas Solids

**DOI:** 10.1002/anie.201908327

**Published:** 2019-08-28

**Authors:** Jan‐Michael Mewes, Paul Jerabek, Odile R. Smits, Peter Schwerdtfeger

**Affiliations:** ^1^ Centre for Theoretical Chemistry and Physics The New Zealand Institute for Advanced Study Massey University Auckland 0632 Auckland New Zealand; ^2^ Mulliken Center for Theoretical Chemistry University of Bonn Beringstr. 4 53115 Bonn Germany; ^3^ Department for Molecular Theory and Spectroscopy Max-Planck-Institut für Kohlenforschung (KOFO) Kaiser-Wilhelm-Platz 1 45470 Mülheim an der Ruhr Germany

**Keywords:** band gap, noble gases, oganesson, radon, superheavy elements

## Abstract

Oganesson (Og) is the most recent addition to Group 18. Investigations of its atomic electronic structure have unraveled a tremendous impact of relativistic effects, raising the question whether the heaviest noble gas lives up to its position in the periodic table. To address the issue, we explore the electronic structure of bulk Og by means of relativistic Kohn–Sham density functional theory and many‐body perturbation theory in the form of the GW method. Calculating the band structure of the noble‐gas solids from Ne to Og, we demonstrate excellent agreement for the band gaps of the experimentally known solids from Ne to Xe and provide values of 7.1 eV and 1.5 eV for the unknown solids of Rn and Og. While this is in line with periodic trends for Rn, the band gap of Og completely breaks with these trends. The surprisingly small band gap of Og moreover means that, in stark contrast to all other noble‐gas solids, the solid form of Og is a semiconductor.

## Graphical abstract

**Breaking rank**: The electronic structure of the noble‐gas solids of neon to oganesson was investigated by means of relativistic density functional theory and many‐body perturbation theory. This revealed band gaps of 7.1 eV and 1.5 eV for the unknown solids of Rn and Og, which means that, in stark contrast to all other noble gases, solid Og is a (short‐lived) semiconductor.

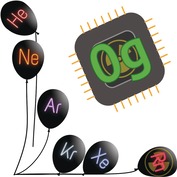

The newly discovered superheavy element oganesson (Og) belongs to the group of noble gases and completes the seventh and presumably last period of the periodic table.^1^ Its only known isotope ^294^Og is rather short‐lived ($0.58_{-0.18}^{+0.44}$ ms)^2^, ^3^ and has been predicted to have some very unusual (atomic) properties for a noble gas.^4^, ^5^, ^6^, ^7^, ^8^, ^9^, ^10^, ^11^ These can be traced back to the presence of two major relativistic effects: (i) the large spin‐orbit separation between the 7p_1/2_ and 7p_3/2_ orbitals with a resulting energy splitting of 10.1 eV,^4^, ^12^ and (ii) the contraction of the vacant 8s orbital. To illustrate these effects, Figure 1 depicts the radial densities as well as energy levels of the valence orbitals of Rn and Og. While the maxima of the densities of the valence s and p orbitals are very close to each other in Rn, the increased spin‐orbit coupling (SOC) in Og drives the 7p_3/2_ maximum towards the 8s orbital. The difference is even more pronounced in the energy regime (inlay of Figure 1). Here the splitting between the 7p_1/2_ and 7p_3/2_ levels in Og is almost as large as the 7s–7p_1/2_ separation, whereas in Rn, the splitting between the 6p levels is much smaller than the respective 6s–6p separation. Lastly, the relativistic contraction of the 8s orbital of Og renders it more compact (*r*_max_=2.41 Å, non‐rel. 3.34 Å) than the 7s orbital of Rn (*r*_max_=2.71 Å, non‐rel. 3.10 Å).

**Figure 1:**
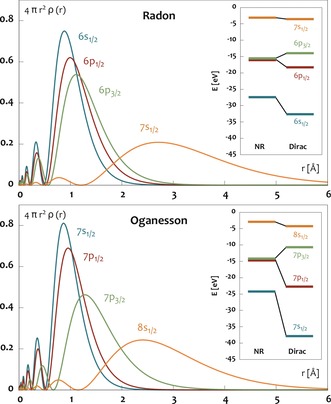
Radial densities and energy levels (in the inset) for the valence orbitals of Rn (top) and Og (bottom) from relativistic (and non‐relativistic) Dirac‐Hartree–Fock calculations for the excited p^5^s^1^ configuration (^3^P_2_ state) using the GRASP program.^13^

In line with these considerations, a recent computational study of atomic Og using the electron‐localization function has revealed the absence of a distinct shell structure reminiscent of an electron gas.^4^ Therefore, one may speculate that in the bulk Og becomes semiconducting or even metallic. This would be in stark contrast to the experimentally known noble‐gas solids,^14^, ^15^ which are insulators with electronic band gaps ranging from 21.51 eV (Ne) to 9.32 eV (Xe).^16^ For these lighter noble gases, the bulk electronic properties are closely related and virtually identical to the respective atomic quantities. For example, the onset of the absorption of the respective bulk solids, also known as the optical band gap (*O_g_*), essentially coincides with the lowest electronic excitation energies of the isolated atoms (Δ*E*) as evident from the green and orange lines in Figure 2. This is a result of the weakly interacting nature of the noble gases,^21^ whose atomic electronic structure remains unaffected in the bulk.

**Figure 2:**
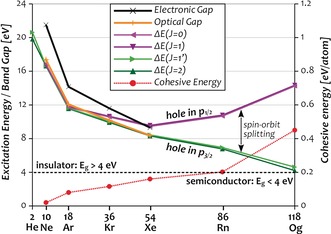
Transition energies (Δ*E*) of the four lowest excited states of the noble‐gas atoms (green: hole in p_3/2_, purple: hole in p_1/2_) compared to optical *O_g_* (orange)^17^ and electronic *E_g_* (black) band gaps^16^ as well as cohesive energies (red, secondary axis)^18^, ^19^, ^20^ of the respective solids. Data for He–Rn from experiment, and for Og (*E*_coh_ also Rn) from coupled‐cluster calculations. See the Supporting Information for details.

Compared to the optical gap *O_g_*, the electronic band gap *E_g_* of the solids is defined by the difference between ionization potential and electron affinity, and thus larger than *O_g_* by the amount of the electron–hole interaction. This quantity, also known as exciton‐binding energy, diminishes from about 5 eV in Ne to about 1 eV in Xe (cf. orange and black lines in Figure 2). While a more detailed discussion of these quantities and their relation for the lighter noble gases is provided in ref. 16, we will focus here on the evolution of Δ*E*, *O_g_*, and *E_g_* with increasing atomic number. By simple extrapolation based on Figure 2, one would anticipate a near‐degeneracy of the three at about 7 eV for Rn, and values just above 4 eV for Og, placing the latter at the borderline between insulators and semiconductors. However, such a simple extrapolation disregards that the close resemblance between atomic and bulk properties may break in the heavier noble gases which are larger, more polarizable and thus interact more strongly. This is reflected in the cohesive energy (*E*_coh_, binding energy of the solid per atom, red line in Figure 2), which increases continuously to 0.23 eV for Rn, and jumps to 0.45 eV for Og.^19^, ^20^ Hence, Og is by far the least noble of the noble gases, less noble even than superheavy copernicium (Cn, *E*_coh_ *=*0.38 eV)^22^, ^23^, ^24^ and thus presumably also a solid at ambient conditions. Accordingly, excitons in solid Og and perhaps also Rn may exhibit a delocalization and stabilization compared to the respective excited states of the atoms, which could cause *O_g_* and *E_g_* to fall well below Δ*E*, breaking with the periodic trends and rendering Og a semiconductor.

To shed some light on the electronic nature of Rn and Og and to put the considerations on a quantitative basis, we calculate their band structure by means of relativistic Kohn–Sham density functional theory (DFT) and many‐body perturbation theory in the form of the *GW* method. Moreover, to pin down the impact of spin‐orbit coupling (SOC), we conduct additional calculations in the scalar‐relativistic (SR) limit and establish the accuracy of the computational protocol by including the experimentally known and theoretically well‐studied lighter congeners Ne–Xe.^25^, ^26^, ^27^, ^28^, ^29^

To set the stage for the discussion of the results, let us briefly review the relation between the electronic band gaps and the eigenvalues *ϵ* of Kohn–Sham DFT, which is a hotly disputed topic.^30^, ^31^ Only recently, it has been established that—at least for solids in periodic calculations—the highest occupied and lowest unoccupied eigenvalues (*ϵ_i_* and *ϵ_a_*) can be identified as ionization potential and electron affinity (*ϵ_i_*=−IP^DFT^ and *ϵ_a_*=−EA^DFT^), and accordingly that their difference defines the electronic band gap *E_g_*.^31^ However, *E_g_* obtained at the DFT level is typically much smaller than the exact band gap, which has been related to various errors and approximations of DFT,^30^, ^31^, ^32^, ^33^, ^34^ a discussion of which is beyond the scope of this work. For our purposes, it is important to recognize that the accuracy of the DFT band gap depends on the functional, and typically improves when climbing Jacob's ladder,^35^ i.e., moving from the local‐density approximation (DFT/LDA),^36^ to gradient‐corrected functionals like PBE,^37^, ^38^ to hybrid functionals that contain nonlocal exchange (nlx), like PBE0 (25 % nlx)^39^ or its screened variant HSE06 (25 % nlx at shortranges).^40^ Also the recently introduced meta‐GGA SCAN has been shown to provide band gaps superior to other functionals of the same rank,^41^ and will thus be employed here in addition to HSE06 and PBE.

To systematically improve DFT band gaps irrespective of the functional, it is required to move from the (local) mean‐field picture of KS‐DFT to a correlated many‐body theory such as the *GW* approach. Its name originates from the mathematical form of the electron–electron interaction, which in *GW* theory appears as the product of the one‐particle Green's function *G* and the screened Coulomb potential *W*.^42^, ^43^ Similar to post Hartree–Fock correlation treatments, the approach allows to systematically improve DFT eigenvalues and eigenfunctions, which serve as the starting point for the calculation. Here, we employ a fully self‐consistent variant of *GW*, in which both eigenfunctions and eigenvalues are iterated in the so‐called quasi‐particle approximation.^29^ This has been shown to provide accurate band gaps for the noble‐gas solids Ne and Ar.^28^, ^29^, ^44^

Moving to the results, experimental and calculated band gaps are compiled in Figure 3. Inspection reveals that as expected, DFT affords much too small band gaps which improve slightly with SCAN and HSE06 compared to PBE. However, even with these modern functionals, the predicted band gaps of the noble‐gas solids are more similar to those from DFT/PBE and basic DFT/LDA (not shown)^45^ than to the experimental data. These results demonstrate the fundamental problems in the calculation of band gaps with DFT. In stark contrast, *GW* fully cures the systematic underestimation of DFT and in turn provides remarkable agreement with experimental values as evident from the virtually coinciding orange and black lines in Figure 3. The largest deviation of merely 0.3 eV is obtained for Ne, and the agreement improves even further for the heavier elements with deviations of only 0.07 eV, 0.04 eV, and 0.03 eV for Ar, Kr, and Xe, respectively. The values for Ne and Ar are moreover consistent with previous theoretical investigations.^28^ At the same numerical precision (7^3^ *k*‐points, 128 included bands) the band gaps for Rn and Og attain values of 6.64 eV and 1.00 eV. Accounting for *k*‐point convergence and extrapolating to an infinite number of included bands as described in the Supporting Information provides theoretical best estimates of 7.1±0.5 eV for Rn and 1.5±0.6 eV for Og. However, it bears pointing out that such an extrapolation worsens the agreement for the lighter noble gases and thus probably also for Rn and Og, which is why the error bars are chosen to include both values. Note that the *GW*/PBE results also depend on the size of the employed valence space. A detailed discussion of these technical aspects is provided in the Supporting Information.

**Figure 3:**
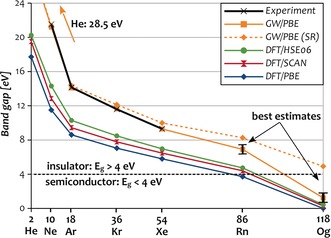
Experimental and calculated electronic band gaps *E_g_* of the noble‐gas solids. Calculations at the DFT (PBE, SCAN and HSE06, dark colors) and *GW* levels (*GW*/PBE, orange). Dotted lines show scalar‐relativistic *GW* results. Numerical values and experimental references are provided in the Supporting Information (Table SII).

Irrespective of these issues, these calculations clearly place Rn within the insulators as is typical for noble gases and in line with the expectations for a weakly interacting system. Og, in contrast, turns out to be a semiconductor with a band gap well below the atomic excitation energy, meaning there is a delocalization and in turn stabilization of excitons in bulk Og. Hence, in contrast to the lighter noble‐gas solids, Og cannot be classified as a weakly interacting system.

To rationalize the surprisingly large decrease of the band gap between Rn and Og, it is instructive to compare the band structures as well as the impact of SOC for Xe, Rn, and Og compiled in Figure 4. Apart from the much smaller band gap, the band structure of Og along the l‐Γ‐X path is very similar to Rn and Xe. Like all noble‐gas solids, Og exhibits a direct band gap located at the Γ point. As expected, the conduction band is dominated by s character, and the valence band is dominated by p character. SOC causes the highest, at the scalar‐relativistic level doubly degenerate band to split as indicated. The resulting additional band is dominated by p_1/2_ character and exhibits by far the largest stabilization in Og. The effect of SOC on the energy of p_3/2_ levels is comparably smaller. The total magnitude of the splitting of Og at the Γ‐point is in excellent agreement with the value derived from atomic IPs of 10.1 eV.^4^ The impact of SOC onto the size of the band gaps is reflected most accurately in the results of the SR and SO relativistic *GW*/PBE calculations compiled in Figure 3 (dotted vs. solid blue lines). A notable deviation can be observed starting at Kr, for which the difference between the SR and SO levels amounts to merely 0.5 eV. Moving on to the heavier noble gases, the value continuously increases to 0.7 eV for Xe and 1.5 eV Rn and eventually jumps to 3.7 eV for Og. This closely resembles the evolution of the cohesive energies and clearly shows that while spin‐orbit effects have mainly a qualitative influence on band gaps for all elements up to and including Rn, they become game‐changing for Og, where they alone almost make the difference between insulator and semiconductor.

**Figure 4:**
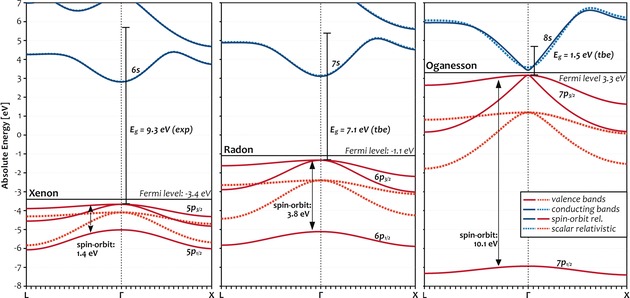
DFT/SCAN band structures of Xe, Rn, and Og along the L‐Γ‐X symmetry‐path (42 points) at the spin‐orbit (SO) relativistic (darker solid lines) and scalar‐relativistic (SR, lighter dotted lines) levels of theory using the 8 (Xe) and 18 (Rn, Og) electron valence spaces. The (SO‐DFT) Fermi level is depicted by a black line. Its SO/SR values are Xe: −3.40/−3.72 eV, Rn: −1.09/−2.14 eV, Og: 3.30/1.50 eV). Arrows and lines depict the spin‐orbit splitting of the valence bands (DFT/SCAN) as well as experimental (exp) and theoretical best estimates (tbe) for the band gaps (at the Γ‐point) to scale.

In summary, band gaps of the noble‐gas solids have been calculated at the spin‐orbit and scalar relativistic levels with DFT and the *GW* method. While bands gaps are systematically underestimated at the DFT level, *GW* calculations provide very accurate band gaps for the experimentally known noble‐gas solids from Ne to Xe. For Rn and Og, theoretical best estimates of 7.1±0.5 eV and 1.5±0.6 eV are provided, revealing that while solid Rn is an insulator similar to its lighter congeners, solid Og breaks with the periodic trend and turns out to be a semiconductor. The reason for the surprisingly sharp decline of the band gap between Rn and Og was eventually traced back to the strong spin‐orbit splitting of the valence 7p shell. While the reported semiconducting nature of Og may help to guide the interpretation of future atom‐at‐a‐time adsorption experiments,^24^, ^46^ the band gap of solid Rn is experimentally accessible, as evident from the measurement of its melting point more than 100 years ago.^47^

Regarding the correlation between the lowest atomic transition energies and band gaps discussed in the introduction, it appears that up to and including Rn the band gaps of Group 18 closely follow the trend in the atomic excitation energies, whereas the band gaps of Og are much below the atomic transition energies. The breakdown of this correlation indicates a delocalization and stabilization of excitons in bulk Og, which is absent in all of its lighter congeners, demonstrating that Og is indeed a very unusual Group 18 element and does not adhere to the classical picture of an inert noble gas. However, while breaking with well‐established group trends, Og appears to be a typical member of the seventh period, the most prototypical aspect of which may be just that: the breaking of group trends.

## Computational Methodology

DFT and *GW* calculations were carried out with VASP 5.4.4.^48^, ^49^, ^50^, ^51^ Experimental structures were used for Ne to Xe,^52^, ^53^, ^54^, ^55^ and for Rn and Og high‐level computational structures were employed (cf. Table SII, Supporting Information).^20^ The core region was modeled using the projector‐augmented wave (PAW) approach of Joubert and Kresse with the potentials for He to Rn taken from the VASP library.^56^, ^57^ For Og, for which no PAWs are available, new ones were created using the same basic structure as for Rn.^22^ Further details concerning the DFT and *GW* calculations are provided in the Supporting Information.

## Conflict of interest

The authors declare no conflict of interest.

## Supporting information

As a service to our authors and readers, this journal provides supporting information supplied by the authors. Such materials are peer reviewed and may be re‐organized for online delivery, but are not copy‐edited or typeset. Technical support issues arising from supporting information (other than missing files) should be addressed to the authors.

Supplementary
