# Supplementary material for: Oganesson Is a Semiconductor: On the Relativistic Band‐Gap Narrowing in the Heaviest Noble‐Gas Solids
Source: Angew Chem Int Ed Engl. 2019 Aug 28;58(40):14260–4. doi: 10.1002/anie.201908327 (PMC6790653; doi:10.1002/anie.201908327)
Supplement: Supplementary file 1 — Supplementary [file ANIE-58-14260-s001.pdf]

Angewandte Chemie
Eine Zeitschrift der Gesellschaft Deutscher Chemiker

Supporting Information

Oganesson Is a Semiconductor: On the Relativistic Band-Gap Narrowing in the Heaviest Noble-Gas Solids

Jan-Michael Mewes, Paul Jerabek, Odile R. Smits, and Peter Schwerdtfeger*

anie_201908327_sm_miscellaneous_information.pdf

Supporting Information

In this supplementary material more information is provided about the computational details, the generation and testing of PAW potentials for Og, as well as on the calculation of the theoretical best estimates. Input and output files of all shown VASP calculations (including POTCARs) can be provided upon request from one of the authors (JMM). POTCARs are also available for download in the supplementary material of ref. 1.

Details on Employed PAW Potentials – Core electrons are modeled using the projector-augmented wave (PAW) approach of Joubert and Kresse.[2,3] The respective POTCAR files containing the PAW parameters are taken from the VASP library for He−Rn. For He−Xe, the Element_GW POTCARS are used, which have 2 valence electrons (VE) for He and 8 VE for Ne. For Xe, Rn and Og, most calculations employ the intermediate Rd_d_GW POTCARs which additionally include the semi-core d^{10} shell (18 VE). Here, we additionally consider the PAWs with the largest valence-space available (Element_sv_GW POTCARs) which also include the semi-core s and p shells (26 VE, $6s^2 6p^6 6d^{10} 7s^2 7p^6$ valence space). For Og, for which no POTCAR files are available in the library, new POTCARs were devised using the same basic structure as for Rn (PAW approach, PBE all-electron calculation, 18 and 26 VE).

PAW generation and evaluation for Og – This was accomplished using the pseudo-potential (PP) generation package of VASP and the PBE functional for the atomic all-electron calculation.[4,5] Since the POTCAR generation package of VASP only allows for a scalar-relativistic treatment of the valence space, which leads to unacceptable errors for the spin-orbit splitting of the $7p$ shell in Og, the parameters for POTCAR were further refined to match the one-particle energies of the immediate valence space ($7p, 8s$) from an atomic calculation with VASP to a four-component 4c-PBE/dyall.d-aug-ae3z reference calculation conducted with DIRAC-17.[6] This was particularly successful for the 18VE PAW, which in turn provides excellent agreement with the 4c-PBE all-electron reference for the one-particle energies (absolute $\Delta E_{7p,8s} \approx 0.1$ eV, relative $\Delta\Delta E_{7p-8s} = 0.03$ eV, *cf.* also Table I), as well as a reasonable agreement for the distance in the Og dimer ($r_e^{\mathrm{AE}} = 4.37$ Å, $r_e^{18\mathrm{VE}} = 4.34$ Å). In further tests, both the 18 and 26 VE POTCARs (in combination with the SCAN, PBEsol and PBE-D3BJ functionals) were shown to accurately reproduce the structural parameters, bulk moduli and cohesive energies obtained from a many-body expansion based on relativistic coupled-cluster results.[7]

Small *vs* large valence space PAWs – Although the small-core 26 VE PAW provides slightly improved bulk properties and a better dimer distance ($r_e^{26\mathrm{VE}} = 4.38$ Å) for Og, the agreement for the one-particle energies with the all-electron reference is significantly worse. In particular the spin-orbit splitting of the $7p$ level, which has been found to be critical for the band gap, is too small with the 26 VE PAW, as evident from the too high energy of the $7p_{1/2}$ level (*cf.* Table I). Accordingly, also the $7p_{1/2} - 7p_{3/2}$ splitting of solid Og at the GW/PBE and DFT/SCAN levels is significantly smaller with 7.5 eV and 7.0 eV, compared 12.2 eV and 10.1 eV with the 18 VE PAWs (all at the Γ-point), and the latter values are in better agreement with the high-level reference of 10.1 eV.[17] Taken together with the distinctly better agreement of the *GW*/PBE/8VE calculation with the experimental value for Xe compared to the *GW*/PBE/26VE value (*cf.* Table II and Figure 3 main article), which becomes even worse if more bands are included (+0.8 eV for 256 bands), this leads us to the conclusion the smaller valence spaces provides more accurate band gaps for Xe−Og. Whilst this needs to be investigated further, we exclusively focus on the results obtained with the smaller valence space in the main article, and consider the large-core results here for the sake of completeness. Apart from that, the most important outcome of this investigation, namely that Rn is an insulator and Og a semiconductor, is the same for the band gaps obtained with the 26 VE PAWs.

TABLE I. Comparison of the single-particle (valence-orbital) energies of the Og atom calculated at the 4c-PBE/dyall.d-aug-ae3z (AE) level of theory to the values obtained at the spin-orbit relativistic PBE level with VASP using the 18 VE and 26 VE PAW potentials.

Orbital	AE	18VE (Δ)	26VE (Δ)
$7s$	−27.08	−27.48 (−0.40)	−28.74 (−1.66)
$7p_{1/2}$	−14.69	−14.61 (+0.08)	−11.22 (+3.47)
$7p_{3/2}$	−5.49	−5.40 (+0.09)	−5.62 (−0.13)
$8s$	−1.37	−1.26 (+0.11)	−1.35 (−0.02)

Details on *GW* calculations *GW* calculations are conducted in the quasi-particle approximation iterating energies as well as wavefunctions and including diagonal and off-diagonal elements as implemented in VASP 5.4.4. (ALGO=QPGW). The reported values are the result of five GW iterations, after which the band gaps are reasonably converged (last step $\Delta E_g < 0.02$ eV). Starting point for the GW iterations are tightly converged DFT/PBE (and DFT/SCAN) calculations. Surprisingly, the better performance of SCAN in the DFT formalism does not translate into a better agreement at the *GW*/SCAN level. Instead, the band gaps are very systematically too large by about $1-2$ eV, showing almost exactly the same evolution along the group as *GW*/PBE. The *GW*/SCAN results are therefore not considered in the main article. For (Ne−Xe) we use experimental and for Rn and Og high-level theoretical structures. Using DFT/SCAN or DFT/PBE-D3BJ optimized structures yields very similar results ($\Delta E_g < 0.1$ eV) except for Rn, where the

TABLE II. Calculated and available experimental structural parameters, electronic band gaps E_g (in eV, at the Γ-point) and lowest atomic excitation energies ΔE (in eV) of the Ne−Og. Scalar-relativistic values are set in parentheses (GW and PBE only), and calculations with the larger 26 electron valence-space are marked 26VE. = Note that part of the differences between the SR and spin-orbit relativistic GW calculations for the lighter elements are due to a technical issue, rendering them too large. These differences are more accurate at the DFT/PBE level. Experimental data for electronic band gaps taken from.[8] The value for Ne varies (21.4 − 21.7 eV) depending on the source,[9,10] see ref. 11 for an overview. Atomic excitation energies ΔE (in eV) for Ne to Xe are from experiment,[12] and from FS-CCSD calculations for Rn and Og.

	R_0	E_g						ΔE	
Atom	exp (calc)	exp	GW (SR)	GW 26VE (SR)	PBE (SR)	SCAN	HSE06	exp	calc
Ne	3.1564^a	21.51	21.20 (21.36)	–	11.50 (11.54)	12.84	14.32	16.610	-
Ar	3.7478^b	14.15	14.08 (14.35)	–	8.61 (8.67)	9.44	10.29	11.548	-
Kr	4.009^c	11.61	11.64 (12.13)	–	7.04 (7.27)	7.80	8.48	9.915	-
Xe	4.3358^d	9.32	9.29 (9.99)	9.85 (10.58)	5.80 (6.23)	6.47	6.98	8.315	8.390
Rn	(4.415)^e	-	6.90 (8.37)	7.33 (8.83)	3.71 (4.82)	4.40	4.74	6.772	6.769
Og	(4.396)^e	-	1.22 (4.92)	2.37 (5.66)	< 0.01 (3.28)	0.28	0.44	-	4.205

^{a} Ref. 13. ^{b} Ref. 14. ^{c} Ref. 15. ^{d} Ref. 16. ^{e} Estimated from relativistic coupled-cluster calculations.[7]

influence is somewhat larger with 0.4 eV.

In general, all calculations are conducted with a k-point grid of 6^3, and only the final GW/PBE calculations are conducted with a finer 7^3 grid. Exploratory calculations for Og and Ne with an 8^3 grid indicate convergence with respect to the number of k-points (ΔE_g < 0.02 eV). Already the difference between 6^3 and $7^3 k$-points is small (< 0.03 eV) for all elements but Og, for which it amounts to about 0.1 eV. The energy cutoff is set at 300 eV, where exploratory calculations with 400 eV for Rn and Og indicate convergence (ΔE_g < 0.01 eV). The lowest 128 bands are included for He−Xe, and the lowest 256 bands for Rn and Og. Exploring convergence with respect to the number bands with the reduced $6^3 k$-point grid shows that including up to 512 bands has a notable influence of 0.1 − 0.2 eV on the band gaps of Rn and Og. However, since inclusion of this many bands with the highest k points grid leads to prohibitively expensive calculations, we include their influence in the best estimates by extrapolating to an infinite number of included bands.

Theoretical best estimates for band gaps – Our theoretical best estimates constitute a compromise between the convergence of the level of theory on one hand, and on the other the observation that for the lighter congeners, not the highest but a specific level provided the best agreement with experimental data, namely GW/PBE with 128 included bands and the smaller valence space. Hence, to obtain balanced theoretical best estimates, we focus on the calculations with the smaller valence space, explicitly include the convergence of k-points and number of included bands, but set the error-bars such that the 1σ-range just includes the results obtained with 128 bands (Rn 6.64 eV, Og 1.00 eV). Doing so leads to the final estimates of 7.1 ± 0.5 eV for Rn and 1.5 ± 0.6 eV for Og.

Atomic excitation energies – The transitions characterizing the atomic excited states displayed in Figure 2 are He: ${}^1S_0(1s^2) \rightarrow {}^3S_1/{}^1S_0(1s^1 2s^1)$, Ne−Og: ${}^1S_0(p^6) \rightarrow {}^3P_{0,1,2}/{}^1P_1(p^5 s^1)$. To obtain atomic excitation energies, we used the Fock-space coupled cluster module including single and double excitations (FS-CCSD) of DIRAC-17,[6,19] utilizing the X2C$_{\text{mmf}}$ Hamiltonian with the Gaunt term included together with the dyall.a4cv basis set .[20,21] In the coupled cluster calculations, all electrons and virtual orbitals up to 816.3 eV (30.0 a.u.) were included in the correlation treatment. The results are summarized in Table IV in comparison to numerical Dirac-Hartree-Fock-Breit (DHFB) results obtain from a modified version of program GRASP to

TABLE III. Convergence of the band gaps of Rn and Og with respect to k-points, energy cut-off and number of bands included in the GW calculations. All results obtained with the smaller 18 VE PAWs. Extrapolation to infinite bands corresponds to the x-intersection of a plot of the last three values against $1/(N_b^2)$.

Level	Xe	Rn	Og
K666, E300, N_b **128**	9.26	6.64	0.88
K666, E300, N_b **196**	–	6.80	1.02
K666, E300, N_b **256**	9.50	6.90	1.10
K666, E300, N_b **384**	–	7.01	1.23
K666, E300, N_b **512**	–	7.09	1.27
extrapol.(256,384,512)	–	**7.14**	**1.33**
experiment	9.30	–	–
**K666**, E300, N_b 256	9.26	6.90	1.10
**K777**, E300, N_b 256 (main)	9.27	6.90	1.22
**K777**, E300, N_b 128	9.26	6.73	0.86
**K888**, E300, N_b 128	–	–	0.88
K666, **E300**, N_b 196	–	6.78	1.02
K666, **E400**, N_b 196	–	6.78	1.01

TABLE IV. Atomic excitation energies $^1S_0[np^6] \rightarrow J[np^5(n{+}1)s^1]$ (in eV). Other results: For Xe and Rn experimental results from Ref. 12, for Og calculated CIPT results from Lackenby *et al.* are taken.[18]

Atom	J-level	DHFB +QED	FS-CCSD +Gaunt	FS-CCSD +Gaunt+QED	other
Xe	2	9.60	8.39	8.40	8.32
	1	9.72	8.54	8.54	8.44
	0	10.91	9.66	9.66	9.45
	1	10.97	9.75	9.76	9.57
Rn	2	6.33	6.77	6.78	6.77
	1	6.52	6.98	6.98	6.94
	0	10.22	10.88	10.89	[10.66][a]
	1	10.29	10.89	10.90	[10.79][a]
Og	2	4.64	4.20	4.22	4.20[b]
	1	5.16	4.62	4.63	4.55[b]
	0	15.44	14.24	15.44	-
	1	15.51	14.35	15.51	-

[a] Estimated experimental value. [b] CIPT results from Lackenby et al..[18]

account for QED effects .[22,23] In both cases QED effects were taken into account from a perturbative treatment at the DHF level using the self-energy effective operator of Flambaum and Ginges and the vacuum polarization from an Uehling potential plus higher order terms (Källèn-Sabry) corrections.[24] We notice that if spin-orbit coupling becomes large, many other transitions from the valence $p_{3/2}$ shell into vacant nlj shells become energetically more favorable compared to transitions out of the valence $p_{1/2}$ shell. This already happens for Rn,[12] and even more so for Og.[18]

[1] Trombach, L.; Ehlert, S.; Grimme, S.; Schwerdtfeger, P.; Mewes, J.-M. Exploring the Chemical Nature of the Super-Heavy Main-Group Elements by Means of Efficient Plane-Wave Density-Functional Theory. *Phys. Chem. Chem. Phys.* **2019**, –.

[2] Blöchl, P. E. Projector Augmented-Wave Method. *Phys. Rev. B* **1994**, *50*, 17953.

[3] Kresse, G.; Joubert, D. From Ultrasoft Pseudopotentials to the Projector Augmented-Wave Method. *Phys. Rev. B* **1999**, *59*, 1758.

[4] Perdew, J. P.; Burke, K.; Ernzerhof, M. Generalized Gradient Approximation Made Simple. *Phys. Rev. Lett.* **1996**, *77*, 3865.

[5] Perdew, J. P.; Burke, K.; Ernzerhof, M. Erratum: Generalized Gradient Approximation Made Simple. *Phys. Rev. Lett.* **1997**, *78*, 1396.

[6] DIRAC, a relativistic ab initio electronic structure program, Release DIRAC17 (2017), written by L. Visscher, H. J. Aa. Jensen, R. Bast, and T. Saue, with contributions from V. Bakken, K. G. Dyall, S. Dubillard, U. Ekström, E. Eliav, T. Enevoldsen, E. Faßhauer, T. Fleig, O. Fossgaard, A. S. P. Gomes, E. D. Hedegård, T. Helgaker, J. Henriksson, M. Iliaš, Ch. R. Jacob, S. Knecht, S. Komorovský, O. Kullie, J. K. Lærdahl, C. V. Larsen, Y. S. Lee, H. S. Nataraj, M. K. Nayak, P. Norman, G. Olejniczak, J. Olsen, J. M. H. Olsen, Y. C. Park, J. K. Pedersen, M. Pernpointner, R. di Remigio, K. Ruud, P. Sałek, B. Schimmelpfennig, A. Shee, J. Sikkema, A. J. Thorvaldsen, J. Thyssen, J. van Stralen, S. Villaume, O. Visser, T. Winther, and S. Yamamoto (see `http://www.diracprogram.org`).

[7] Jerabek, P.; Smits, O. R.; Mewes, J.-M.; Peterson, K. A.; Schwerdtfeger, P. Solid Oganesson via a Many-Body Interaction Expansion Based on Relativistic Coupled-Cluster Theory and from Plane-Wave Relativistic Density Functional Theory. *The Journal of Physical Chemistry A* **2019**, *123*, 4201–4211.

[8] Runne, M.; Zimmerer, G. Excitonic Excitations and Desorption from Rare-Gas Solids. *Nuclear Instruments and Methods in Physics Research Section B: Beam Interactions with Materials and Atoms* **1995**, *101*, 156–168.

[9] Klein, M.; Venables, J. *Rare Gas Solids, Vol. 1*; Academic Press: New York, 1976.

[10] Schwentner, N.; Himpsel, F. J.; Saile, V.; Skibowski, M.; Steinmann, W.; Koch, E. E. Photoemission from Rare-Gas Solids: Electron Energy Distributions from the Valence Bands. *Phys. Rev. Lett.* **1975**, *34*, 528–531.

[11] Bernstorff, S.; Saile, V. Experimental Determination of Band Gaps in Rare Gas Solids. *Opt. Commun.* **1986**, *58*, 181 – 186.

[12] Moore, C. *Atomic Energy Levels*; Natl. Stand. Ref. Data Ser., Natl. Bur. Stand.: USA, 1971.

[13] Batchelder, D. N.; Losee, D. L.; Simmons, R. O. Measurements of Lattice Constant, Thermal Expansion, and Isothermal Compressibility of Neon Single Crystals. *Phys. Rev.* **1967**, *162*, 767–775.

[14] Peterson, O. G.; Batchelder, D. N.; Simmons, R. O. Measurements of X-Ray Lattice Constant, Thermal Expansivity, and Isothermal Compressibility of Argon Crystals. *Phys. Rev.* **1966**, *150*, 703–711.

[15] Losee, D. L.; Simmons, R. O. Thermal-Expansion Measurements and Thermodynamics of Solid Krypton. *Phys. Rev.* **1968**, *172*, 944–957.

[16] Sears, D. R.; Klug, H. P. Density and Expansivity of Solid Xenon. *J. Chem. Phys.* **1962**, *37*, 3002–3006.

[17] Jerabek, P.; Schuetrumpf, B.; Schwerdtfeger, P.; Nazarewicz, W. Electron and Nucleon Localization Functions of Oganesson: Approaching the Thomas-Fermi Limit. *Phys. Rev. Lett.* **2018**, *120*, 053001.

[18] Lackenby, B. G. C.; Dzuba, V. A.; Flambaum, V. V.

Atomic Structure Calculations of Superheavy Noble Element Oganesson ($Z = 118$). *Phys. Rev. A* **2018**, *98*, 042512.

[19] Visscher, L.; Eliav, E.; Kaldor, U. Formulation and Implementation of the Relativistic Fock-Space Coupled Cluster Method for Molecules. *J. Chem. Phys.* **2001**, *115*, 9720–9726.

[20] Sikkema, J.; Visscher, L.; Saue, T.; Iliaš, M. the Molecular Mean-Field Approach for Correlated Relativistic Calculations. *J. Chem. Phys.* **2009**, *131*, 124116.

[21] Dyall, K. G. Relativistic Quadruple-Zeta and Revised Triple-Zeta and Double-Zeta Basis Sets for the 4p, 5p, and 6p Elements. *Theor. Chem. Acc.* **2006**, *115*, 441–447.

[22] Dyall, K.; Grant, I.; Johnson, C.; Parpia, F.; Plummer, E. GRASP: A General-Purpose Relativistic Atomic Structure Program. *Comput. Phys. Commun.* **1989**, *55*, 425 – 456.

[23] Thierfelder, C.; Schwerdtfeger, P. Quantum Electrodynamic Corrections for the Valence Shell in Heavy Many-Electron Atoms. *Phys. Rev. A* **2010**, *82*, 062503.

[24] Fullerton, L. W.; Rinker, G. A. Accurate and Efficient Methods for the Evaluation of Vacuum-Polarization Potentials of Order $Z\alpha$ and $Z\alpha^2$. *Phys. Rev. A* **1976**, *13*, 1283–1287.
